# Functional outcome of multiple toe transfer procedure for open multiple transmetacarpal amputation secondary to a crushing injury: A case report

**DOI:** 10.1016/j.ijscr.2024.110774

**Published:** 2024-12-26

**Authors:** Viet Tan Nguyen, Van Doan Le

**Affiliations:** Upper extremity trauma and Microsurgery Department, 108 Military Central Hospital, 1 Tran Hung Dao Street, Hanoi, Viet Nam

**Keywords:** Toe transfer, Transmetacarpal amputation, Amputation, Metacarpal hand, Case report

## Abstract

**Introduction:**

Reconstruction for open multiple transmetacarpal amputation secondary to a crushing injury is really challenging. Some treatment approaches could be proposed. To avoid the drawbacks of a prosthesis and hand transplantation such as the high cost, and long-term side effects of anti-rejection drugs, toe transfers were chosen.

**Presentation of CASE:**

A 33 year-old man suffered a crushing injury that led to a left transmetacarpal amputation and a right arm amputation. The left hand was reconstructed by toe transfers to restore the thumb, index and middle fingers. After 75 months of follow-up, on the left hand, the patient could recover tripod pinch. Tripod pinch strength was 4 pounds, grip strength was 10 pounds. Static 2 points discrimination of the reconstructed thumb, index, and middle fingers was 12, 13, and 15 mm, respectively. The maximum grip span was 8 cm. With the left reconstructed hand, the patient could return to almost all activities in daily life and work.

**Discussion:**

For this case, toe transfers seems to be more suitable and effective than other methods. Tripod pinch reconstruction provides stability, a strong grip, a wide working-distance, and a good appearance.

**Conclusion:**

This case highlights the efficacy of tripod pinch reconstruction by toe transfers in addressing complex transmetacarpal amputation, providing effective solution even when the contralateral upper extremity was also amputated.

## Highlights

• Reconstruction for open multiple transmetacarpal amputation secondary to a crushing injury is really challenging.
• Some treatment approaches could be proposed such as a prosthesis, hand transplantation, and toe transfers. In comparison with reconstructed hand, a prosthesis gives less functional outcomes and rates of prosthetic rejection among upper limb amputees are high. The main disadvantage of hand transplantation is long-term side effects of anti-rejection drugs.
• After careful consultation with the patient and family, toe transfers to reconstruct the tripod pinch were chosen.
• On long-term follow-up, the patient was able to return almost activities in daily life and work. Tripod pinch strength was 4 pounds, grip strength was 10 pounds. The maximum grip span was 8 cm.

## Graphical abstract

**Figure:**
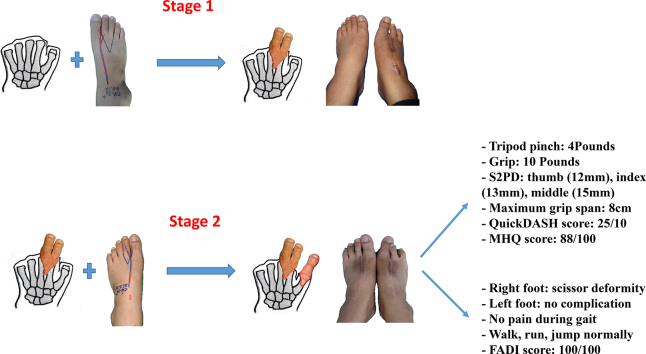

## 1 Introduction

Open multiple transmetacarpal amputation (metacarpal hand type 2) secondary to a trauma is devastating and challenging [[1], [2], [3], [4]]. There are some treatment approaches for this injury such as a prosthesis, hand transplantation, and toe transfers. Each method has different profits and drawbacks. In comparison with reconstructed hand, a prosthesis gives less functional outcomes and rates of prosthetic rejection among upper limb amputees are high [5]. The advantage of hand transplantation is restoring a new tissue with a similar structure, but the disadvantages are difficulty in finding a donor and long-term side effects of anti-rejection drugs [6]. To avoid the disadvantages of the two above methods, toe-to-hand transfers would be chosen. In this article, we present an extraordinary outcome of toe transfers in a case sustaining not only a left transmetacarpal hand but also a right arm amputation.

The following case report has been reported in line with the SCARE criteria [7].

## 2 Case presentation

A 33 year-old man suffered a crush injury that resulted in a left metacarpal hand type 2 A (transmetacarpal amputation of all 4 fingers with thumb amputation at the proximal phalange base) and a right arm amputation. Five months later, in November 2017, he was referred to our hospital. Hand and arm transplantations were planned for him, however, it was so difficult to find a donor, and the last, toe transfers to reconstruct the tripod pinch of the left hand were chosen. Preoperatively, the patient was fully informed of the procedure's profits and drawbacks via pictures, videos, and results of our similar cases and other studies. Clay models were used to help the patient understand the aesthetic results after the surgery.

**Fig. 1:**
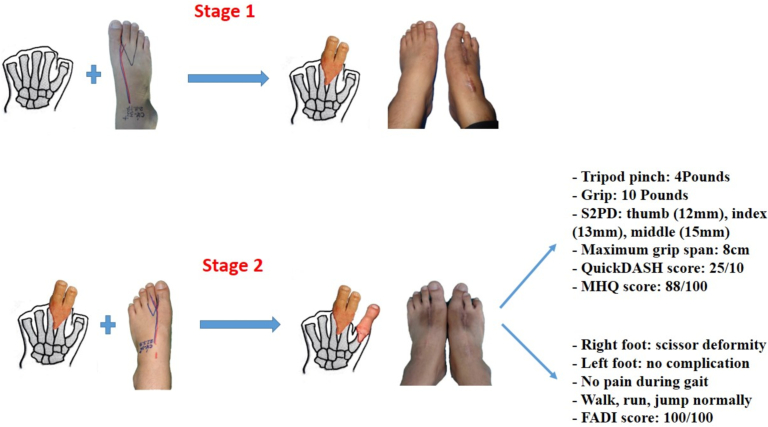
Study flow diagram. Abbreviations: S2PD, Static 2 points discrimination; QuickDASH: Quick Disabilities of the Arm, Shoulder, and Hand; MHQ: Michigan Hand Outcomes Questionnaire; FADI: Foot and Ankle Disability Index.

**Fig. 2:**
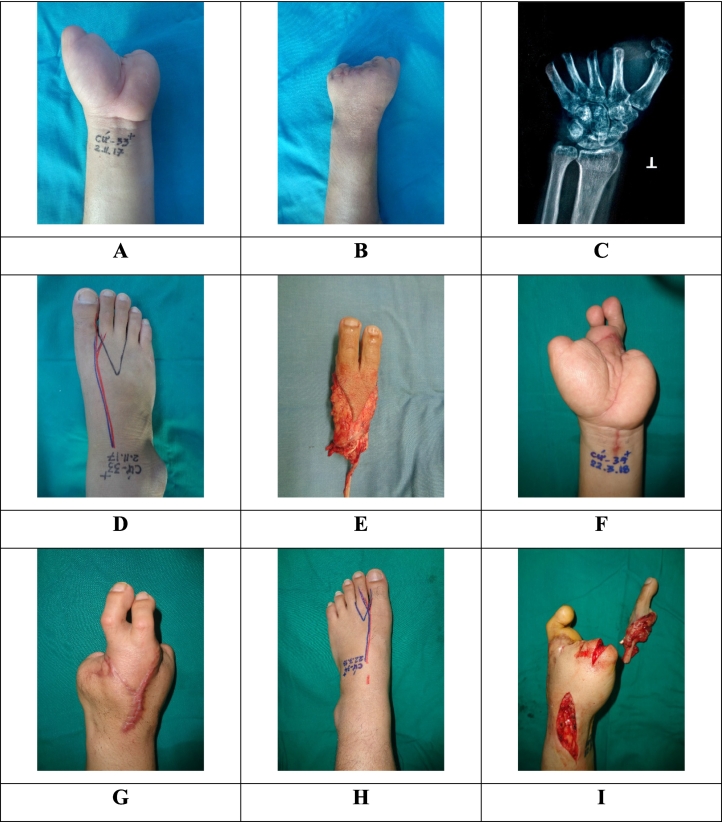
Images of surgical technique. A-E: stage 1 (the left index and middle fingers were reconstructed first with combined second and third toes from the right foot). F-I: stage 2 (the thumb was then reconstructed with the left second toe).

Surgical technique: the left hand was reconstructed in two stages (Fig. 1). The left index and middle fingers were reconstructed first with combined second and third toes (including the metatarso-phalangeal joints) from the right foot. The thumb was then reconstructed, 4 months later, with the left second toe. (Fig. 2A-I). Bone fixations were performed by cerclage wiring technique. The flexor, extensor tendons and nerves of toes were sutured to the corresponding tendons stumps and nerve stumps of the hand, respectively. In both toe-to-hand transfer surgeries, the radial vascular bundle and cephalic vein were used as recipient vessels.

**Fig. 3:**
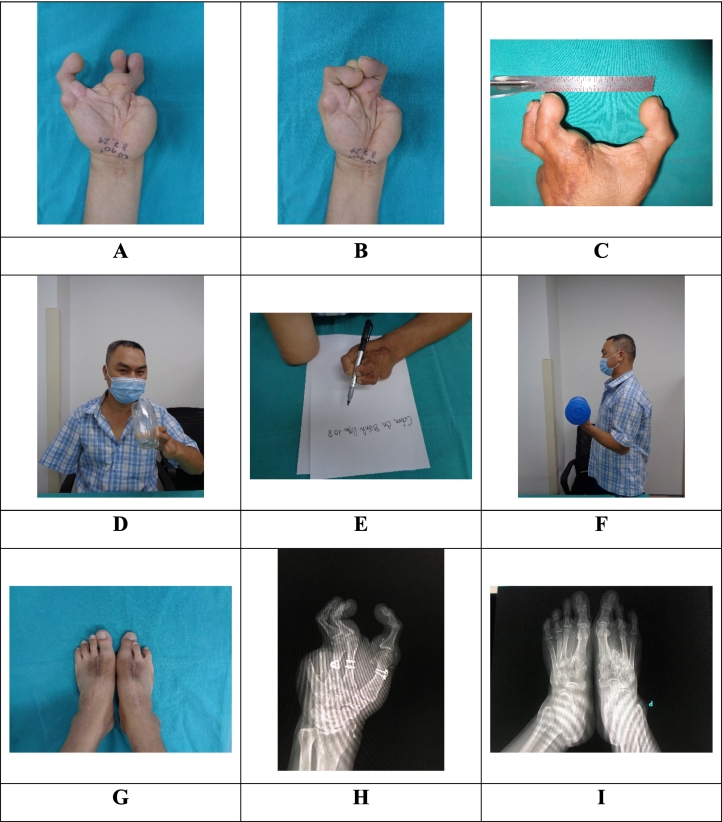
Secondary results.

Results: All transferred toes survived without complications. Secondary results (after 75 months of follow-up): in the left hand, the patient could recover tripod pinch (Fig. 3A-I). Tripod pinch strength was 4 pounds, grip strength was 10 pounds. Static 2 points discrimination of the reconstructed thumb, index, and middle fingers was 12, 13, and 15 mm, respectively. The maximum grip span was 8 cm. His Quick Disabilities of the Arm, Shoulder, and Hand score was 25/100. The Michigan Hand Outcomes Questionnaire score was 88/100 (The score of overall hand function, activities of daily living, pain, work performance, aesthetics, and patient satisfaction was 85, 73, 86, 0, 88, and 100, respectively). The patient could return to almost all activities in daily life and work like eating (Video 1), writing (Video 2), exercising (Video 3), and cycling (Video 4).

As to the donor site, although the right foot had scissor deformity (migration of the fourth toe toward the great toe), the patient did not have permanent pain during gait. When wearing shoes, he could walk, run, jump, go up and down stairs normally (Video 5). The foot and ankle disability score was 100/100.

Despite changing job from a worker to a seller, he was very satisfied with the surgical outcomes and refused to wear a prosthesis or more surgery to reconstruct the right arm by transplantation.

## 3 Discussion

A transmetacarpal amputation combining an arm amputation of the contralateral side in the same patient is devastating and really rare in the population [4,8]. In our case, three treatment approaches were supposed, including a prosthesis, hand transplantation, and toe transfers. The best solution for metacarpal hand remains unclear [9,10]. In the medical literature, we have not found any studies comparing outcomes between these three methods. In our case, it was difficult to find a donor and after careful consultation, toe transfers were chosen. Our case proves the efficacy of toe transfers in dealing with a metacarpal hand, giving good outcomes even when a contralateral arm was lost.

The main goal in the reconstruction of metacarpal hand type II according to Wei was to restore a tripod pinch because this method provides a stronger grip, more stability, a better appearance, and a wider working-distance than a pulp-to-pulp pinch [[1], [2], [3]]. Contrary to Wei's recommendation, Venkatramani et al. (2016) advocated restoring only 2 digits to achieve pulp-to-pulp pinch, therefore the morbidity on the donor foot would be less [11]. In our case, we supported Wei's recommendation because our patient also had an amputation of the right arm. Wei's approach was later supported by many authors because of its benefits [[12], [13], [14], [15]].

The exact location of reconstructed long digits was still a controversial issue. According to Wei, reconstruction of the ulnar fingers (ring and little) was suitable for patients whose jobs require a power grip and a large hand span. Reconstruction of the radial fingers (index and middle) was the best for patients requiring fine manipulation [1,16,17]. In the case of transferring a single digit, the preferred location for digit reconstruction by Venkatramani was the index finger [11]. In our clinical case, we found that restoring pinch strength was more important than grip strength, so we advocated the reconstruction of the index and middle fingers.

In the donor foot, the limitations of this case were the decrease of the metatarsal arch and the scissor deformity of the right foot. To prevent the scissor deformity in combined second and third toe transfers, Wei proposed to harvest a limited dorsal and plantar skin flaps extending only to the midpoint of the first and digital web spaces, thus allowing for direct donor-foot closure. To eliminate potential gait disturbances, the plantar metatarsal arch should be maintained by avoiding metatarsal shaft osteotomies or reconstructed by bone grafting if necessary [18]. However, our patient did not feel any exact pains in his feet and could perform almost daily living activities. In comparison with the results received in hand, the foot morbidities were trivial and un-significant.

Another disadvantage of our method was the bad aesthetic of the neohand. However, after a long time of surgery, the patient was very happy and felt confident with the appearance of his left hand. The aesthetic and satisfied scores (according to Michigan Hand Outcomes Questionnaires) were 88/100 and 100/100, respectively. These results once again confirmed the role of preoperative counseling.

The advantages of toe transfers for this case include (1) restoring a good functional hand, (2) lower cost compared to a prosthesis, (3) not need to find a donor and depend on anti-rejection medicines postoperatively. The disadvantages are (1) the ugly appearance of the neohand, (2) the morbidity of the donor foot.

## 4 Conclusion

Multiple transmetacarpal amputation is challenging, especially in case combining an arm amputation of the contralateral side. Toe transfers to reconstruct a tripod pinch is an effective approach to restore hand function and help the patient to return to daily activities and work rapidly.

The following are the supplementary data related to this article.

Video 1 Eating Video 1

Video 2 Writing Video 2

Video 3 Exercising Video 3

Video 4 Cycling Video 4

Video 5 Running Video 5

## Author contribution

Viet Tan Nguyen: follow-up and post-operative management, data collection, data analysis, manuscript drafting.

Van Doan Le: performing the operations, follow-up and post-operative management.

## Consent

Written informed consent was obtained from the patient for publication of this case report and accompanying images. A copy of the written consent is available for review by the Editor-in-Chief of this journal on request.

## Ethical approval

The study was approved by our research committee.

## Guarantor

Viet Tan Nguyen

## Research Registration Number

Not applicable for a case report.

## Funding

None.

## Conflict of interest statement

None.
